# Biological activated carbon filter for greywater post-treatment: Long-term TOC removal with adsorption and biodegradation

**DOI:** 10.1016/j.wroa.2021.100113

**Published:** 2021-08-13

**Authors:** Angelika Hess, Eberhard Morgenroth

**Affiliations:** a Eawag: Swiss Federal Institute of Aquatic Science and Technology, 8600 Dübendorf, Switzerland; b ETH Zürich, Institute of Environmental Engineering, 8093 Zürich, Switzerland

**Keywords:** Biological activated carbon, Greywater reuse, Adsorption, Biodegradation, Backwashing

## Abstract

Biological activated carbon (BAC) filters can be used to remove residual total organic carbon (TOC) from greywater after a membrane bioreactor. The two main TOC removal processes are adsorption to the granular activated carbon (GAC) and biological degradation. Biodegradation leads to the growth of microorganisms in the filter bed, which can lead to increased pressure loss over the filter bed. However, the roles of sorption and biodegradation in long-term TOC removal and how they complement each other are unclear. We monitored TOC removal from greywater in a BAC filter installed following a membrane bioreactor over more than 900 days. Removal performance depended on the operational time of the BAC filter, the influent TOC concentration, and in the upper part of the filter on the empty bed contact time (EBCT). Across the overall filter, the EBCT did not significantly influence TOC removal, showing that the filter was sufficiently large for the range of flow rates observed. Analysis of the long-term data revealed the equal importance of sorption and biodegradation over the whole operation period and the whole filter bed. Most of the TOC was removed in the upper part of the filter, where biodegradation was the dominant mechanism. In the lower part of the filter, sorption capacity remained and allowed high influent TOC concentrations to be buffered. The generous filter design with low average filtration rates ensured long-term TOC removal. The only maintenance needed was backwashing, which was required only after more than 800 days of operation. Backwashing effectively reduced the pressure loss but had no significant influence on the effluent water quality. Our study shows that BAC filters are a suitable post-treatment step for the treatment of greywater with highly variable flow and TOC concentrations.

## Highlights

• Total organic carbon removal in a biological activated carbon filter for greywater post-treatment monitored over 900 days.
• Long-term stable total organic carbon removal was demonstrated with around 60% removed.
• Biodegradation and adsorption overall contributed equally to the total organic carbon removal.
• Remaining sorption capacity at the bottom of the filter buffered high influent total organic carbon.
• Little reactor maintenance required: only infrequent backwashing.

## Graphical abstract

**Figure:**
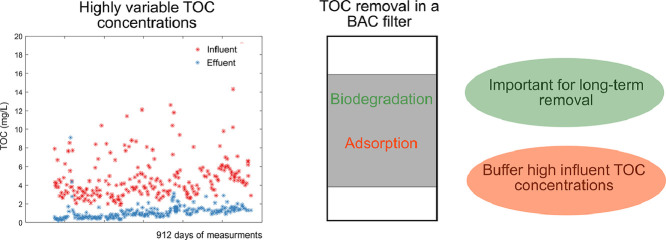

## 1 Introduction

Greywater reuse offers substantial water-saving potential with the benefit that the water is also available during dry periods (National Academies of Sciences 2016). To ensure safe reuse of water for applications involving direct contact with humans, such as showers and hand-washing, pathogens need to be inactivated (Shi et al., 2018). Reducing the organic carbon to low levels is important i) to prevent regrowth of pathogens during storage and distribution (Prest et al., 2016), ii) to ensure reliable disinfection performance (Winward et al., 2008), and iii) to prevent the negative aesthetic effects of carbon degradation after treatment (Dietrich 2006). Analysing long-term data and understanding how TOC degradation is influenced by different factors in a greywater treatment system treating real greywater are therefore important.

The two main removal mechanisms for organic carbon in biological activated carbon (BAC) filters are adsorption and biodegradation. For fresh granular activated carbon (GAC), adsorption is the main removal mechanism. As the volume of water treated increases, adsorption capacity is exhausted, bacteria accumulate in the filter bed, and biodegradation becomes increasingly important for TOC removal. From a certain point of time on, biodegradation is the main removal mechanism (Simpson 2008). So far, the extent to which various depth segments in the BAC filter may be at different stages of organics removal has not been studied, and neither has the extent to which biodegradation is beneficial to maintaining sorption capacity. Various pilot and lab studies have been conducted to study factors influencing removal performance in BAC filters. Temperature, empty bed contact time (EBCT), and backwash frequency have been shown to impact the removal performance (Emelko et al., 2006, Hallé et al., 2015). Yet, how a BAC filter may be designed to use both adsorption and biodegradation optimally for TOC removal has yet to be clarified.

Optimal design and operation of BAC filters require an understanding of the proportions of TOC that are removed by two processes, biodegradation and adsorption. Two main approaches are taken to estimating the proportions of the two processes. The first approach is to inhibit biodegradation, for example by adding sodium azide, autoclaving the GAC, or using fresh GAC without biomass (Islam et al., 2016, Rattier et al., 2012, Sharaf and Liu 2021, Zhiteneva et al., 2020). The second approach is to stop sorption by using either exhausted GAC or material without sorption capacity, such as anthracite (Greenstein et al., 2018, Liang et al., 2003). Fundneider et al., (2021b) used long-term pilot-study data to estimate that biodegradation removes 25–42% of the organics. Smolin et al., (2020) proposed a quantitative model to assess the contributions of biodegradation, sorption, and bioregeneration to TOC removal in a BAC filter. In this study, we used long-term data from a building-scale BAC filter to estimate the proportions of sorption and biodegradation and confirmed the results with batch tests with and without GAC. An additional challenge is that sorption and biodegradation are not always readily separable because they can also interact (Herzberg et al., 2003).

Biological degradation of organic carbon in the filter leads to bacterial growth. This biomass can either accumulate in the filter or be washed out from the filter (Velten et al., 2011). Accumulated biomass and inorganic particles can clog the biofilter and lead to increasing pressure loss over the filter bed. Regular backwashing is considered important to maintaining long-term performance and preventing exhaustive pressure loss (Piche et al., 2019). However, the extent to which backwashing is necessary is unclear when the influent to the BAC filter is free of inorganic particles that might clog the filter and biomass is the main cause of pressure loss. Depending on the influent concentration of organics, the ratio of biomass to TOC degraded can vary (Eiler et al., 2003). The organic carbon that is not used for biomass production is oxidized to CO2 by microbes to gain energy for maintaining cell function. Long-term performance in the BAC filter with little pressure loss is better maintained with low biomass production.

In this study, TOC removal was monitored over a filter bed for more than 900 days in a BAC filter treating greywater after a membrane bioreactor (MBR). The real greywater was highly variable in TOC concentration; this allowed us to test the filter's performance over a wide range of influent concentrations. Our study estimated the influence of various operational parameters on long-term removal performance. A carbon mass balance over the filter bed was used to estimate sorption and biodegradation and the extent to which accumulation of biomass on the filter bed hindered operation. We assessed the influence of i) a generous BAC filter design, ii) reactor maintenance, and iii) backwashing on the reactor's performance in providing stable effluent water quality.

## 2 Material and methods

### 2.1 Experimental system

**Fig. 1:**
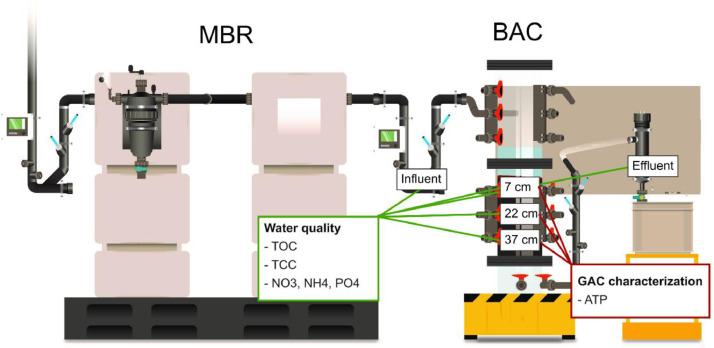
Greywater treatment system in the Water Hub at NEST. An MBR is followed by a BAC filter for post-treatment. This study analysed the treatment performance of the BAC.

In the Water Hub at NEST, source separated wastewater streams are treated separately (https://www.eawag.ch/en/department/eng/projects/water-hub/, accessed 26.03.2021). The light greywater collected in the building is treated with an MBR (adapted version from Aquacell 800, newterra) followed by a custom-made BAC filter (Fig. 1). The MBR degrades organic carbon and nitrifies ammonium to nitrate and the BAC filter removes residual organic carbon to provide biologically stable water (Table 1). We treated on average 330 L/d (0 to 1155 L/d) of greywater coming from a fitness unit, public bathrooms, and bathrooms in flats. The BAC filter was operated in gravity-driven down-flow mode, and we used Calgon Filtrasorb 400 GAC (characterization of Calgon Filtrasorb 400, e.g., in Morlay and Joly 2010). Sampling ports at 7, 22, and 37 cm depth allowed samples to be taken from various depths for water and GAC analysis. A S*chmutzdecke* with biomass developed on top of the filter bed. To evaluate the contribution of the *Schmutzdecke*, water samples in the GAC bed around 0.5 cm below the *Schmutzdecke* were taken with a glass syringe and a steel needle.

**Table 1:** Water quality across the treatment system. Values are median with 25 and 75 percentiles in parentheses. pH was measured online at a time resolution of 4–5 s.

	NH_4_-N (mg/L)	NO_3_-N (mg/L)	PO_4_-P (mg/L)	pH (-)
Raw greywater (n = 71)	1.3 (0.5, 5.3)	2.3 (1.3, 3.1)	0.2 (0.2, 0.5)	7.7 (7.4, 8.0)
After MBR (n = 246)	0.02 (0.02, 0.3)	9.8 (6.6, 14.0)	1.4 (0.9, 2.4)	8.1 (7.9, 8.3)
After BAC (n = 285)	0.02 (0.02, 0.04)	10.1 (7.0, 14.1)	1.4 (0.7, 2.0)	7.6 (7.5, 7.7)

The highly variable greywater production in the building and the operation of the MBR led to intermittent flow into the BAC filter with periods with stagnant water. The filter has an inner diameter of 0.3 m, and the filter bed is 0.52 m deep. The mean EBCT was 78 min (SD: 27 min) and the mean filtration rate 0.42 m/h (SD: 0.45 m/h). The system was operated for 912 days.

#### 2.1.1 Backwashing

The filter was backwashed once on day 836, towards the end of the experimental period. The backwash was performed with tap water in up-flow mode with a flow of 20 L/min, corresponding to an up-flow velocity of 17 m/h, for a duration of 11 minutes. A 1 L sample of the backwash water was taken every minute in autoclaved glass bottles. The backwash water was analysed for TOC, TCC, ATP, and TSS. The maximum bed expansion during the backwash was 14%.

#### 2.1.2 Pressure loss

Pressure loss over the filter bed was determined with two pressure sensors, one located above the filter bed and the other below the filter bed. Both sensors were of the Cerabar T PMC131 (Endress+Hauser) type, and measurement values were saved every 4–5 s.

### 2.2 Measurements

#### 2.2.1 Total organic carbon

Total organic carbon (TOC) was sampled in glass vials twice a week and only at times with flow through the BAC filter. Samples were stored in darkness at 4°C until analysis. Due to the pandemic, from day 668 to day 730, samples were taken once a week and were stored up to 10 weeks at -20°C until analysis. TOC was measured with a total organic carbon analyser (Shimadzu TOC-L, Kyoto, Japan). To assess the uncertainty of the sampling and the measurement, influent and effluent samples were measured in triplicate once. The standard deviation was below 1% of the measurement value for both influent and effluent.

#### 2.2.2 Total cell concentration

Total cell concentration (TCC) was measured with a CytoFLEX (Beckman Coulter, Brea, California, USA) at a flow rate of 60 µL/min for 60 s. TCC was measured with SYBR Green I stain. All samples were measured in triplicate.

#### 2.2.3 ATP

The biomass was quantified with the method described in (Velten et al., 2007) and as reported previously (Hess et al., 2020). In short, 1 g wet weight of GAC particles was transferred in a muffled glass vial with 10 mL of 0.2 µm filtered (Millex-GP, Merck Milipore Ltd) tap water. The sample was sonicated for 3 min (Bandelin Sonorex), 9 mL supernatant was collected in a separate muffled glass vial, and the sample was refilled with 9 mL of 0.2 µm filtered (Millex-GP, Merck Milipore Ltd) tap water. This step was repeated three times in total. The collected supernatant was vortexed, and 0.5 mL of the sample was incubated at 38°C for at least 4 minutes. Some 100 µL of sample together with 100 µL of BacTiter-Glo™ Microbial Cell Viability Assay (Promega Corporation, Madison, WI, USA), were measured for relative light units (RLU) and converted to ATP concentrations.

#### 2.2.4 Batch growth

To assess the biological stability of the water samples, 20 mL of 0.2 µm filtered (Millex-GP, Merck Milipore Ltd) sample was inoculated with 20 µL of unfiltered sample, incubated at room temperature (22 ± 1°C) for 5 d in 40 mL glass vials, and agitated. Afterwards, the samples were analysed for TCC. All glassware used was muffled at 450°C for 4 h. All samples were taken in triplicate.

### 2.3 Batch TOC degradation tests

Batch tests were conducted to evaluate the influence of remaining sorption and of different types of biomass on the TOC removal. Four batches were compared:

i) Biomass in suspension: water sample taken from the influent of the BAC filter with biomass in suspension;
ii) Detached biomass: biomass detached by manual shaking from the GAC into 0.2 µm filtered (polycarbonate Nuclepore membrane filter, Whatman) influent water;
iii) *Schmutzdecke*: biomass sampled with a steel needle and a glass syringe from the *Schmutzdecke* of the BAC filter;
iv) Biomass on GAC: biomass with exhausted GAC from the sampling at 7 cm depth of the BAC filter.

To obtain comparable results, the same amount of biomass (quantified with ATP) was added to each batch: 1 L of the biomass in suspension, 1 g of GAC with biomass, detached biomass from 2 g of GAC, or 3 mL of *Schmutzdecke*. For all the samples except suspension, the BAC filter influent water was 0.2 µm filtered (polycarbonate Nuclepore membrane filter, Whatman) and added to provide the same nutrients to all batches. The samples were placed in an overhead shaker at room temperature for 5 days, and TOC and TCC samples were taken on days 1, 2, 4, and 5. All samples were done in duplicate and the glassware used was autoclaved.

### 2.4 Data analysis

#### 2.4.1 Linear regression model

The long-term TOC data was analysed with a linear regression to estimate the relationship between TOC_out_/TOC_in_ and various input variables. We looked at three independent input variables: days of operation of the filter (*time*), influent TOC concentration (*TOC_in_*), and empty bed contact time (*EBCT*) (Equation 1).

(1) $$\frac{TOC_{out}}{TOC_{in}}=b_{0}+b_{1}\cdot time+b_{2}\cdot TOC_{in}+b_{3}\cdot EBCT$$

The linear regression was performed with the *fitlm* Matlab command (The MathWorks, Inc). The validity of the model was checked by looking at the distribution of the residuals. The model was used as a data analysis tool but cannot be used to predict future removal performances of the BAC filter.

#### 2.4.2 Mass balance TOC removal

**Fig. 2:**
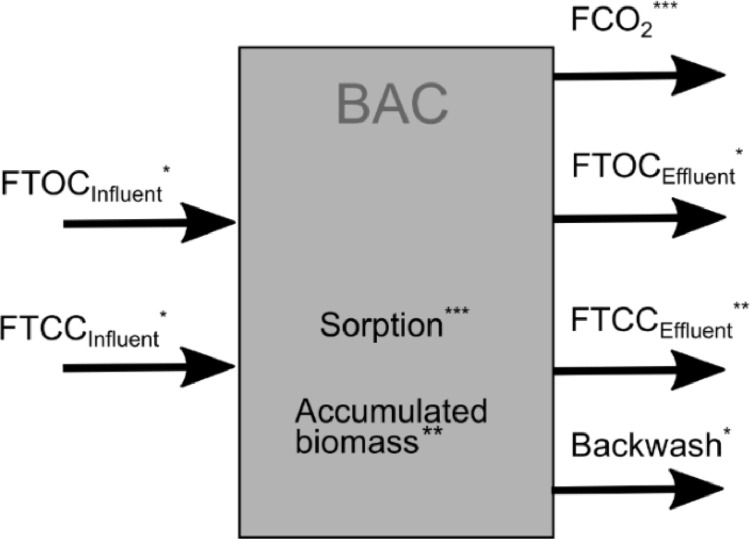
Organic carbon loads considered for the mass balance. For the influent and effluent water, TOC (FTOC) and TCC (FTCC) loads were considered. FTCC is part of FTOC, but FTCC is also measured separately for the analysis of the biomass. FCO_2_ is the biodegraded carbon that is not assimilated as biomass. *: calculated from measured variables, **: calculated with measured variables and some assumptions, ***: calculation based on major assumptions.

A mass balance was performed with the long-term data to better understand the TOC removal. The following paragraphs present the assumptions and calculations for the TOC mass balance that differentiates between the two removal processes. Fig. 2 and Table 2 give an overview of the loads considered and the main assumptions for the calculations. Afterwards, the calculations for the different loads are explained in more detail.

(2) $$FTOC_{biodegraded}=FTOC_{removed}-FTOC_{sorption}$$

(3) $$FTOC_{CO_{2}}=FTOC_{biodegraded}-FTOC_{assimilated}$$

(4) $$FTOC_{assimilated}=FTOC_{cells,accumulated}+FTOC_{cells,washedout}+FTOC_{cells,backwash}$$

**Table 2:** Measurements and assumptions made for the calculations of the TOC mass balance over the BAC filter. *: calculated from measured variables, **: calculated with measured variables and some assumptions, ***: calculation based on major assumptions.

Removal category	Measured variables for calculation		Assumptions	Uncertainty assessed
Removed organic carbon*	TOC_Influent_, TOC_Effluent_	2/week		
	Flow	continuous		
Sorption^⁎⁎⁎^	TOC_Influent_, TOC_Effluent_	2/week	Peak influent on day 67 and subsequent desorption determines the maximum amount of TOC sorbed, q_e_	Different assumptions to calculate q_e_
	M_GAC_	constant		
	Flow	continuous		
Biodegradation^⁎⁎^	-	-	Calculated with equation (2)	
CO_2_^⁎⁎⁎^	-	-	Calculated with equation (3)	
Assimilated biomass^⁎⁎^	-	-	Calculated with equation (4)	
Accumulated biomass^⁎⁎^	ATP_GAC_	After backwashing	ATP/cell	Different ATP/cell values
	M_GAC_	-	TOC/cell	
Washed out biomass^⁎⁎^	TCC_Influent_, TCC_Effluent_	Infrequent	TOC/cell	TCC_Effluent_ additionally estimated based on turbidity
	Flow	continuous		
Backwashed biomass*	TCC_Backwash,_	1/minute	TOC/cell	
	Flow_Backwash_	Constant		

Details of the calculations are shown in Supporting Information S3.

##### 2.4.2.1 Flow

Flow was calculated from the pressure measurement on top of the filter bed as described in (Hess et al., 2021). For the mass balance, a resolution of 1 min was used.

##### 2.4.2.2 TOC

To calculate the overall TOC load (FTOC), each flow value was multiplied with the nearest TOC measurement for both the influent and the effluent of the BAC filter. The total cell load (FTCC) are part of the FTOC, but only constitute around 0.1% of the TOC. We assume that the effluent FTOC does not contain a significant amount of GAC debris during normal operation and therefore do not consider GAC particles in the mass balance.

##### 2.4.2.3 Total cells in influent and effluent

No regular measurements were available for the total cell concentration in the influent and the effluent of the BAC filter; therefore, the mean concentrations were used to calculate the total load. The TCC was 3.3·10^5^ cells/mL (n = 99) for the influent and 8.6·10^5^ cells/mL (n = 82) for the effluent. A carbon content of 2·10^−14^ gTOC/cell was assumed (Velten et al., 2011). In Hess et al., (2021) we demonstrated a strong correlation between TCC and turbidity (R^2^ = 0.84). Therefore, the effluent TCC was estimated based on measured turbidity to get a range of possible TCC loads leading to 3.2·10^6^ cells/mL for the effluent. The difference between effluent and influent cells was used to calculate the quantity of cells washed out from the filter.

##### 2.4.2.4 Cells accumulated in the BAC filter

After the backwash, 0.0686 g ATP accumulated in the BAC filter, calculated from the measurements taken at the three GAC sampling locations (Supporting Information, Fig. S4). The ATP/cell concentration was determined as 1.1·10^14^ cells/g ATP. Velten et al., (2011) calculated a value of 3 ± 1.5·10^−16^ gATP/cell, and our measurement for biomass detached with shaking gave a value of 4.8·10^13^ cells/g ATP. A carbon content of 2·10^−14^ gTOC/cell was assumed (Velten et al., 2011).

##### 2.4.2.5 Backwash

The TCC load (FTCC_Backwash_) removed during the backwash was calculated with the flow during the backwash (20 L/min) and the TCC concentrations measured in the backwash samples. A carbon content of 2·10^−14^ gTOC/cell was assumed (Velten et al., 2011).

##### 2.4.2.6 Sorption

Sorption was estimated based on the long-term TOC data. The equilibrium loading (q_e_) and the total mass of 20 kg GAC in filter were used to calculate the amount of TOC that was removed due to adsorption. The equilibrium loading was calculated with an integral mass balance based on the TOC load removed in the top 7 cm of the BAC filter. After the highly concentrated influent peak on day 67 (131 mg/L TOC), the TOC first adsorbed and then desorbed back to quite stable removal values. Here, we assumed that q_e_ was reached on day 80 after the phase with desorption. We also assumed that until day 80, 35% of the TOC was removed by biodegradation and 65% by sorption. This led to a q_e_ of 25.6 mgTOC/gGAC. To account of the uncertainty of these assumptions, two scenarios were tested: i) all the TOC was removed by sorption, ii) the GAC was already exhausted before the peak. This led to i) q_e_ = 31.5 mgTOC/gGAC and ii) q_e_ = 14.7 mgTOC/gGAC.

## 3 Results

### 3.1 What influenced the TOC removal performance?

**Fig. 3:**
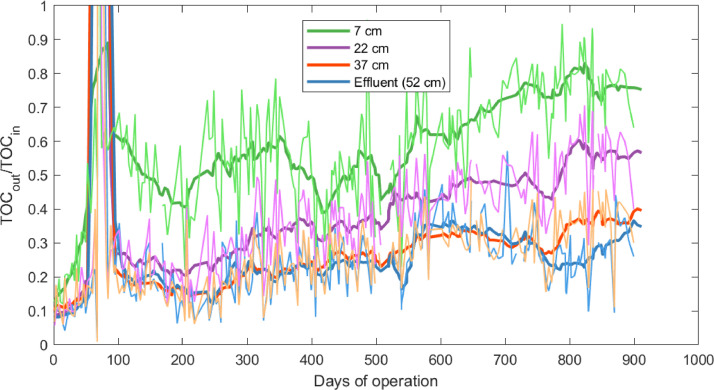
Removed TOC in the building-scale BAC filter over 900 days of operation for the different depths of the filter bed. The bold lines represent the moving averages over 10 measurements.

Long-term TOC removal was monitored over the whole BAC filter and for the different segments of the filter for a period of more than 900 days (Fig. 3, absolute TOC values for influent and the effluents are presented in Supporting Information, Fig. S2). Generally, the effluent concentrations were highest at the first sampling point at 7 cm and then decreased with increasing depth and residence time in the BAC filter. On day 67, an exceptionally high influent TOC concentration of 131 mg/L entered the BAC filter, caused by exceptionally high influent TOC in the raw greywater and insufficient treatment performance of the MBR. When the sample with high influent concentration was taken, the concentration after 7 cm was high (64 mg/L) while it remained low at 22 cm, 37 cm, and in the effluent (2.8, 1.1, and 1.2 mg/L, respectively). Three days later, the TOC influent concentration was back at normal values (8.6 mg/L), while the concentration in the filter were still high with 26, 89, 89, and 43 mg/L at 7, 22, 37 cm, and in the effluent, respectively. Five days after the high influent, the effluent TOC was 4.8, 14, 30, and 36 mg/L for 7, 22, 37 cm, and effluent, respectively. Seven days after high influent, the effluent TOC was 2.2, 3.0, 6.5, and 9.1 mg/L for 7, 22, 37 cm, and effluent, respectively. We could observe how the TOC adsorbed and then with lower influent concentrations desorbed again to values as observed before the peak influent concentrations. After that peak, the moving average for TOC_out_/TOC_in_ varied between 0.4 and 0.8 for 7 cm, between 0.2 and 0.6 for 22 cm, and between 0.1 and 0.4 for 37 cm and the BAC filter effluent.

The long-term TOC data was analysed with a linear regression model to test the independent input variables (Eq. (1)). Only the data after the peak with high influent concentration (after day 140) was used for the linear model.

(1a) $$\frac{TOC_{out}}{TOC_{in}}=b_{0}+b_{1}\cdot time+b_{2}\cdot TOC_{in}+b_{3}\cdot EBCT$$

The estimated coefficients of the linear model for the different depths are listed in Table 3. All the coefficients differed significantly (*p* < 0.05) from 0, except b_3_ for the effluent (marked with an asterisk in the table). Coefficient b_0_ is higher for higher locations in the filter bed. This coefficient shows that after the peak, adsorption was more exhausted in the upper segments than in the lower segments of the filter. Coefficient b_1_ shows that the removal performance deteriorated with time for all segments of the BAC filter. However, this trend was stronger for the segments higher in the filter than for the lower ones. For example, at 22 cm, the TOC_out_/TOC_in_ increased by 0.38 over the 770 days considered. The negative coefficients for b_2_ show that higher influent TOC_in_ decreased TOC_out_/TOC_in_. This effect was in the same range for all segments of the BAC filter. TOC_in_ was on average 4.8 mg/L, ranging from 1.9 mg/L to 19.1 mg/L (data in Supporting Information S4), which thus changed TOC_out_/TOC_in_ by 0.4. Coefficient b_3_ varies for the different segments. Although longer EBCT in the uppermost segment led to lower TOC_out_/TOC_in_, EBCT had no significant influence on the removal performance of the whole BAC filter. The average EBCT of the whole filter was 78 min, ranging from 15 min to 166 min. The flow rate and thus the EBCT depends on the operation of the preceding MBR and the flux through the membrane.

**Table 3:** Estimated coefficients for the linear regression model. Coefficients that are statistically not significantly different from 0 (p > 0.05) are marked with an asterisk (*). More details about the linear model can be found in Supporting Information S2.

	7 cm	22 cm	37 cm	Effluent
**b_0_**	0.61	0.31	0.20	0.23
**b_1_ (y^−1^)**	0.168	0.182	0.117	0.080
**b_2_ (L mg^−1^)**	-0.022	-0.019	-0.016	-0.019
**b_3_ (min^−1^)**	-0.0019	-0.00096	-0.00035	-4.83E-06 *

**Fig. 4:**
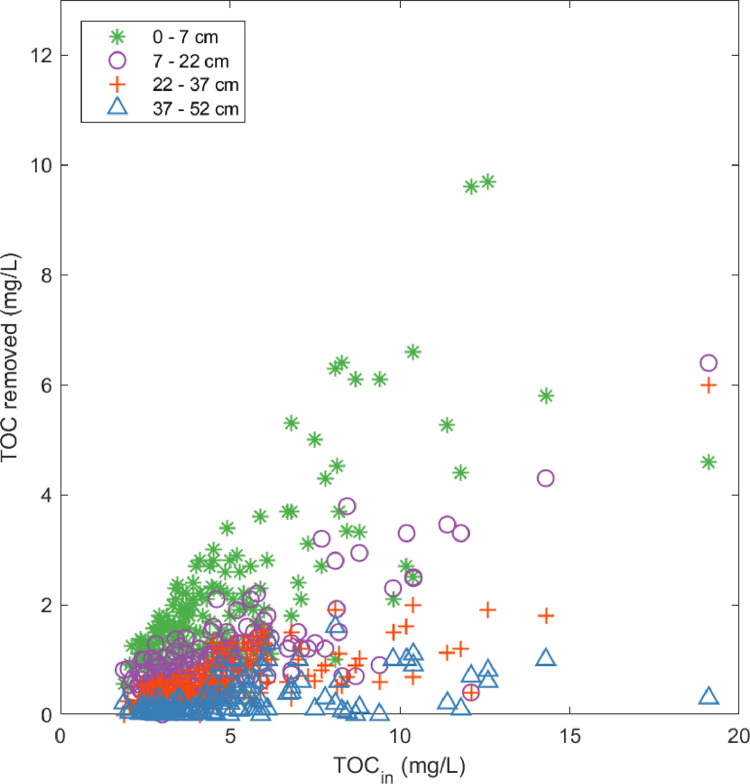
TOC removed in the different segments of the BAC filter for different influent TOC concentrations. For all the segments, the TOC_in_ is the influent into the BAC filter.

Fig. 4 shows the TOC removal in the various segments of the BAC filter for different influent TOC concentrations. The first segment is only 7 cm, whereas the others are 15 cm each and thus have double the GAC volume. The higher the influent TOC, the more TOC is removed in the BAC filter. For all influent concentrations, the highest quantity of TOC was removed in the top 7 cm of the BAC filter. The upper limit of TOC removed in the top segment was defined by a quite clear line with a slope of around 0.85 mgTOC_removed_/mgTOC_in_. The segment from 7 cm to 22 cm behaved similarly to the top segment. For almost all the cases, more TOC was removed in the top segment than in the ones below, and the more TOC was in the influent, the more TOC was removed. For the segment from 22 cm to 37 cm, only once was more than 2 mg/L of TOC removed: for the influent TOC of 19 mg/L. For the segment from 37 cm to 52 cm, the removal was always below 0.4 mg/L up to influent TOC concentrations of 4 mg/L, and with higher TOC_in_, TOC removal rose to 1.6 mg/L. The lowest segment mainly contributed to overall TOC removal for high influent TOC concentrations.

### 3.2 How is the TOC removed?

Over the whole experimental period of 912 days, 1024 g of TOC were removed in the BAC filter. Of all TOC removed, 59% was removed in the top 7 cm, 24% in the second 15 cm, 12% in the third segment, and only 5% in the lowest 15 cm segment of the BAC filter. A few measurements were taken directly below the *Schmutzdecke*. After the *Schmutzdecke*, on average 18% of the incoming TOC (7–36%) had already been removed.

**Fig. 5:**
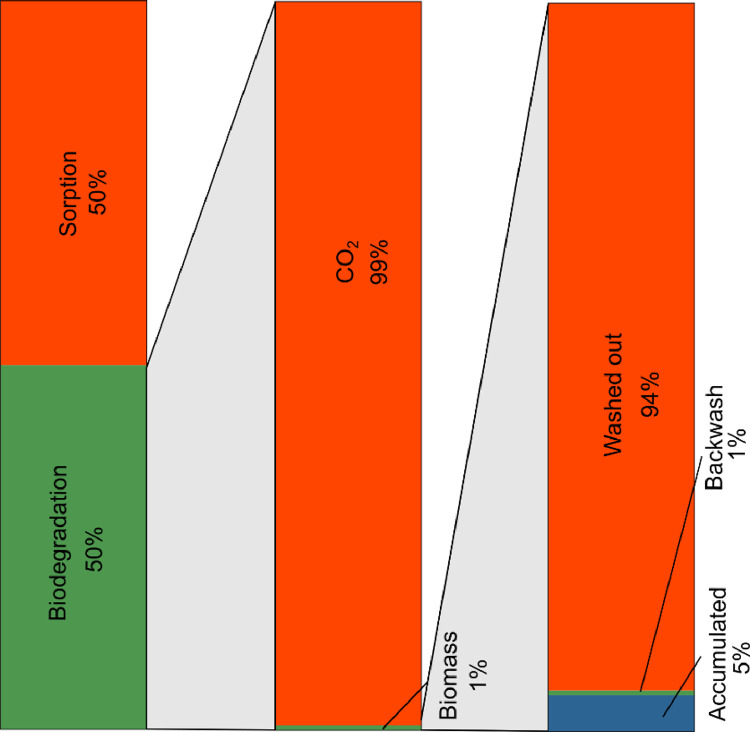
Fate of carbon during 2.5 years of BAC filter operation.

We used a mass balance to estimate that over the whole experimental period and over the whole BAC, 50% of the removed TOC was removed by adsorption onto the GAC, and the other half was removed by biological degradation (Fig. 5). We assumed the equilibrium loading q_e_ for adsorbed TOC to be 25.6 mgTOC/gGAC. We used a range of assumptions to calculate q_e_ to be between 14.7 mgTOC/gGAC and 31.5 mgTOC/gGAC, leading us to estimate the contribution of sorption as between 36% and 77%.

Of the biodegraded carbon, only 1% was assimilated as biomass by accumulation in the BAC filter, washing out, and removal with the backwash, and 99% left the system as CO_2_. Of the biomass that grew in the BAC filter due to carbon degradation, 5% accumulated in the filter bed as biofilm on the GAC, and less than 1% was removed with the backwash on day 836. The remaining 94% of biomass was continuously washed out of the filter with the treated water as suspended solids, leading to higher cell concentrations in the effluent (on average 8.6⋅10^5^ cells/mL) than in the influent (on average 3.3⋅10^5^ cells/mL). The cell concentrations were calculated from infrequent water samples. The calculation of effluent cells from turbidity led to an even higher estimation of cells washed out: 99%. The contribution of carbon that was assimilated in cells increases to 4% (leaving 96% as CO_2_) for this case. A further assumption concerned the cells/ATP ratio, which influences the estimation of the cells accumulated in the filter. The main calculation assumed a value of 1.1⋅10^14^ cells/g ATP, which was measured from biomass removed with sonication from the GAC. With the value we obtained for easily detachable biomass (4.8⋅10^13^ cells/g ATP), the proportion of cells accumulated in the filter decreases to 2%. With a literature value from Velten et al., (2011) of 3⋅10^15^cells/g ATP, which was determined for a BAC filter treating drinking water, the proportion of accumulated cells would increase to 61% of the biomass produced. The quantity of cells in the backwash water show that 1% of the biomass was removed with the backwash. However, the quantity of TOC in the backwash water was 3.4 g, much higher than the TOC in the backwash calculated from TCC (0.02 g TOC). The assumption is that the remaining TOC mainly represents fine GAC particles and dust and is therefore not considered in this TOC mass balance. If the opposite assumption were be made, that all the TOC in the backwash water were related to biomass, for instance extracellular polymeric substances, the proportion of the biomass removed in the backwash would increase to 53%, but 1% of the biodegraded carbon would still be assimilated as biomass.

**Fig. 6:**
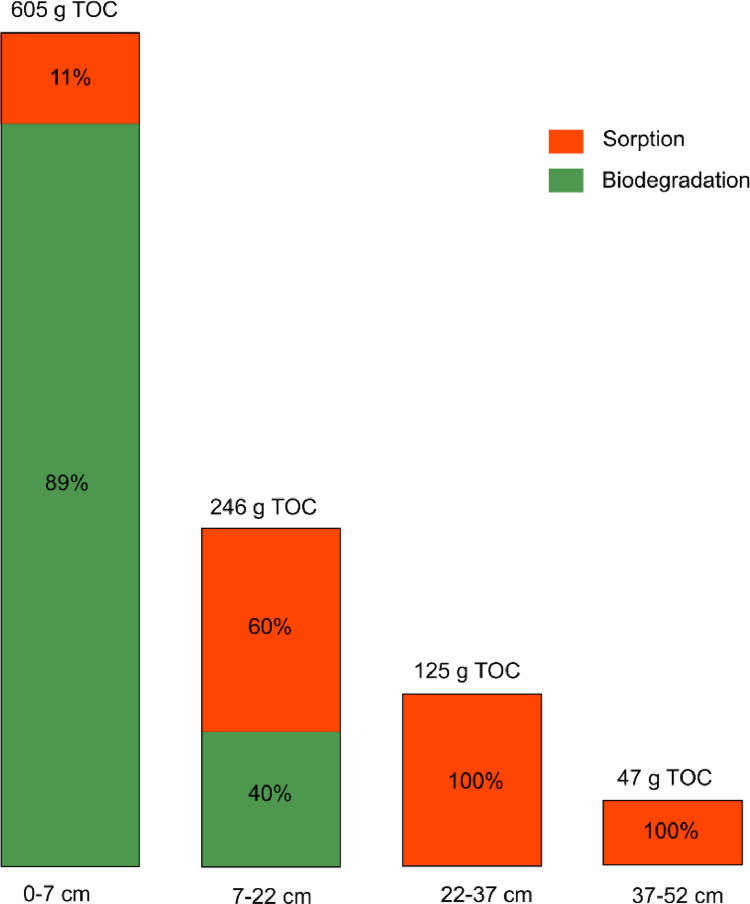
Sorption and biodegradation and the total removed TOC for the different segments of the BAC filter.

Biodegradation and sorption contributed equally to TOC removal in the BAC filter as a whole, but the importance of the two processes varied significantly in the different segments of the filter (Fig. 6). Biodegradation was the main removal mechanism in the top segment of the BAC filter, where it accounted for nearly 90% of the removed TOC. In the top segment, the sorption capacity was exhausted after a few months. For the lower segments, some sorption capacity still remained after more than 900 days of operation. So up to 100% of the carbon could be removed by sorption. Nevertheless, we cannot exclude the possibility that biodegradation also occurred in the lower parts of the filter bed.

We used batch tests with biomass and GAC from 7 cm depths to confirm that sorption did not play a significant role in the top segment of the BAC filter towards the end of the experiment (data shown in Supporting Information S5). In the batch experiments, the same removal performance for biomass from the S*chmutzdecke* and biofilm from GAC was observed for both high initial TOC and for typical influent TOC concentrations. Between 57% and 68% of high initial TOC was removed, and 7–13% of typical influent TOC concentrations. Further, the batch with biomass on GAC did not show improved removal performance compared to batches with only biomass. Additionally, no difference was observed if the biomass was in suspension or in a biofilm.

### 3.3 Influence of backwashing

**Fig. 7:**
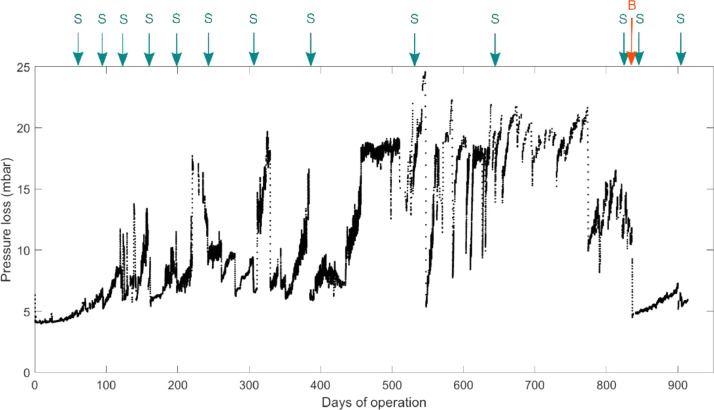
Pressure loss over the filter bed for the whole experimental period of more than 900 days. Arrows indicate events that could have disturbed the filter bed. A single backwash was performed on day 836, indicated by “B”; GAC samplings are indicated by “S”.

The BAC filter was operated without backwashing for more than two years. The backwash performed after 836 days reduced pressure loss over the filter significantly, to values similar to the beginning of the BAC filter operation (Fig. 7). The flow to the BAC filter was controlled to prevent overflow, and thus the inflow was stopped at a pressure loss of 22 mbar. Therefore, pressure losses of more than 22 mbar were prevented. Sudden drops in pressure loss occurred even before the backwash. Some of these pressure loss drops can be explained by reactor maintenance work and sampling of GAC from the filter. However, some of the sudden decreases, such as that on day 455, cannot be explained with the data available.

Although pressure loss over the filter bed was significantly lower after the backwash, backwashing did not influence TOC removal (Fig. 3). Moreover, the backwash did not significantly influence the quantity of bacteria washed out from the BAC filter (data shown in Supporting Information S6). The effluent TCC was 2.0•10^6^ #/mL (SD: 6.8•10^5^ #/mL) before the backwash and 1.9•10^6^ #/mL (SD: 7.1·10^5^ #/mL) after the backwash. Batch growth was measured as an indicator of biological stability (data shown in Supporting Information, Fig. S5). Batch growth in the effluent of the BAC filter was lower directly after the backwash but increased again to prebackwash levels within one day with no mid- to long-term influence on removal performance. The batch growth in the effluent was 7.8·10^5^ #/mL (SD: 3.2·10^5^ #/mL) before the backwash and 8.3·10^5^ #/mL (SD: 3.4·10^5^ #/mL) after the backwash. Both before and after backwash, batch growth was higher at higher locations in the filter bed, showing that the BAC filter was effective in reducing assimilable organic carbon. Further, we assessed the quantity of active biomass in the BAC with ATP. ATP on GAC was reduced directly after backwash and quickly recovered to prebackwash levels, with the highest values at 7 cm (2.4·10^−6^ gATP/gGAC) and lower values at 22 cm (1.3·10^−6^ gATP/gGAC) and 37 cm (5.4·10^−7^ gATP/gGAC) (data in Supporting Information, Fig. S4). Turbidity, which can be used as a proxy for TCC after the BAC filter (Hess et al., 2021), showed that more cells were washed out of the BAC filter than normal after the backwash. However, after around 600 L water volume had passed through the BAC, normal turbidity patterns were observed again.

## 4 Discussion

### 4.1 What influenced the TOC removal performance?

Highly variable flows into greywater treatment systems and the operation of MBRs lead to constantly changing operational parameters such as EBCTs and filtration rates. This study assessed the influence of these changes on TOC removal performance. Operation time influences TOC removal because adsorption capacity is exhausted over time and biological degradation increases. Therefore, removal performance deteriorates fast initially, and then change slows over time (Simpson 2008). Although the TOC breakthrough curve reached the first and the second sampling ports relatively quickly, the change in water quality in the lower segments of the BAC filter was slower. Biodegradation in the upper part allows sorption to continue at the bottom over a long period of operation. Several studies have reported that longer EBCTs improve removal performance up to a certain EBCT but that no further improvement can be achieved if the EBCT is further prolonged (Fundneider et al., 2021a, Seredyńska-Sobecka et al., 2006, Urfer et al., 1997). This fits with the observation reported here that the varying EBCT had a significant influence on removal performance in the upper parts of the filter but not across the overall filter, for which the EBCT was always sufficiently long. One characteristic of decentralized greywater treatment is its highly variable flow (Hess et al., 2020): treatment steps therefore cannot be dimensioned for one specific flow. If good effluent water quality needs to be ensured at all times, a generous BAC filter design with low average filtration rate and long average EBCT is therefore needed to be able to deal with variable flow and thus variable EBCTs. However, compared to main treatment with an MBR, the footprint required and the costs are still low.

High influent TOC concentrations may be caused by either high raw greywater concentrations or low residence times. In either case, less biodegradation occurs in the MBR, leading to higher quantities of biodegradable carbon in the BAC and thus to better removal performances for higher influent TOC concentrations. Furthermore, adsorption equilibria in batch tests also depend on water quality (Yapsaklı et al., 2009). One finding of this study is that for higher TOC_in_ more TOC is removed. This is beneficial for ensuring stable effluent water quality across variable influent concentrations.

The removal performance observed in this study was compared to other long-term studies that used BAC filter for treatment of drinking water after ozonation, greywater treatment, or advanced wastewater treatment. To be able to compare removal performance, we calculated the influent load (Load_in_ = V_treated_/M_GAC_·TOC_in_) for several publications (Supporting Information, Table S1). Of the studies considered, three had a Load_in_ larger than 300 gTOC/kgGAC: this study, Fundneider et al., (2021b), Gibert et al., (2013b). In this study, 41% of the TOC was removed after a filter-bed depth of 7 cm, whereas in Fundneider et al., (2021b) and Gibert et al., (2013b) only around 20% TOC removal were maintained after similar TOC loadings. Although EBCT was higher for Fundneider et al., (2021b) at 25 min, it was comparable between Gibert et al., (2013b) at 13 min and this study at 15 min after 7 cm. EBCT therefore cannot be used as the only explanation for the better removal performance observed in this study. The filtration rate clearly differed across the studies. In this study, the filtration rate was much lower at 0.42 m/h than the 7 m/h in Fundneider et al., (2021b) and the 3.7-9.7 m/h in Gibert et al., (2013b). Typically, EBCT is the main design variable, and the effect of filtration rate is not considered when BAC filters are dimensioned (Urfer et al., 1997). This comparison shows that a low filtration rate potentially helps to maintain higher long-term removal performance.

BAC filters for greywater post-treatment were studied in two other publications: (Ziemba et al., 2020, Zipf et al., 2016). They reported a TOC removal performance of 31% and 50%, respectively. However, it is not clear if this performance would also last long term, because both treated a Load_in_ of less than 50 gTOC/kgGAC. Therefore, direct comparison of their removal performances with this study is not possible.

One specific characteristic of the system in this study is the intermittent flow leading to times with stagnant water. Hess et al., (2020) showed that intermittent flow does not significantly influence removal performance. However, the water source can make an important difference because of the varying composition of the organic carbon (Liang et al., 2007). The literature compared shows that BAC filters tend to work better for greywater post-treatment than for drinking-water treatment. However, more data for long-term performance of BAC filters for greywater treatment is needed.

### 4.2 How is the TOC removed?

The long-term TOC data was used to estimate the proportions of TOC removed by biodegradation and sorption. Overall, 50% of the TOC was removed by biodegradation, but in the top part of the filter, the proportion of biodegradation was higher at 89%. These values for biodegradation are higher than those reported in literature: 26% biodegradation by Sharaf and Liu (2021), 18% by Islam et al., (2016), and 25–42% by Fundneider et al., (2021b). However, the studies vary in the methods used to estimate biodegradation: Sharaf and Liu (2021) and Islam et al., (2016) inhibited biodegradation with sodium azide, whereas this study used long-term data to estimate sorption and biodegradation, which was confirmed with batch tests. Using sodium azide to inhibit biodegradation has two limitations: i) sodium azide does not inhibit all organisms and ii) sodium azide can interact with the GAC and thus influence the sorption capacity (Klimenko et al., 2010). These are two reasons to query whether the sodium azide method may underestimate the proportion of biodegradation and overestimate that of sorption. Another difference is that in this study, the proportion of biodegradation was calculated over long-term removal, whereas Sharaf and Liu (2021) and Islam et al., (2016) examined batch tests before sorption capacity had been reached.

The estimation of sorption in this study also relies on some important assumptions. Our assumption for calculating the equilibrium load on the GAC neglects slower sorption into smaller pores and assumes that it is mainly the surface and the larger pores of the GAC that are relevant to TOC removal (Erdem et al., 2020). Previous research showed that the assumption of constant equilibrium loading is feasible despite variable influent concentration (Hess et al., 2020).

The estimated 1% of organic carbon assimilated in biomass is quite low and shows that a high proportion of biodegraded carbon was used for microbial maintenance. At lower concentrations of organics, lower growth efficiency can be expected, and thus less growth (Eiler et al., 2003). The values calculated in this study are in line with other studies. Velten et al., (2011) estimated that 3% of the removed carbon was assimilated in biomass in a BAC filter treating drinking water. They also estimated that 84% of the biomass was detached and washed out from the biofilter when operated without backwashing. In this study, 94% of the biomass detached continuously, and only a small portion, less than 1% of the biomass, was removed with the backwash. Fundneider et al., (2021b) removed more, around 10% of the biomass, with the backwash but used a different method (volatile suspended solids) to quantify the biomass in the system. They also used water and air for backwashing, while in this study the backwash was performed with water alone.

So far, studies have focussed on biofilm development over the filter bed and how biodegradation replaces adsorption as the main removal mechanism over time (Velten et al., 2011, Gibert et al., 2013a). To our best knowledge, this is the first study that examined the role of sorption capacity remaining at the bottom of the filter in buffering high influent concentrations. Biodegradation was the main removal mechanism in the upper part; it was important to maintaining sorption capacity at the bottom and thus extending the lifespan of the BAC filter. The long-term data was also confirmed by higher sorption capacity for methylene blue at the bottom of the BAC filter (Supporting Information S7). Previous studies have shown that activated carbon has advantages as a biofilm carrier and has a higher biofilm activity than inert media (Herzberg et al., 2003). Here, we showed that an additional advantage of using GAC as a medium in biofilters is the sorption capacity remaining in the bottom part of the filter.

### 4.3 Influence of backwashing

For this system, we found that backwashing reduced pressure loss but did not significantly influence removal performance. This is in line with studies that have shown that biomass quickly returns to prebackwash levels and that effluent water quality is not influenced by backwashing (Gibert et al., 2013a, Piche et al., 2019).

The biomass accumulated in the BAC filter could lead to more microbial decay products in the effluent. Microbial decay products can lead to lower biological stability (Derlon et al., 2014, Velten et al., 2011). This study, found no change in batch growth due to backwashing. This indicates that no significant quantity of old biomass, in which hydrolysis leads to increased batch growth, was removed with the backwash.

During this study, no regular backwashing was necessary. One of the causes of filter clogging is the accumulation of inorganic particles in the BAC filter. In our setup, a membrane with a pore size of 0.04 µm in front of the BAC filter retained the particles and therefore less clogging occurred. Fundneider et al., (2021b) also found this effect of membranes in parallel-operated BAC filters. In both water treatment and decentralized greywater treatment, membranes are becoming increasingly important, and a BAC filter can be used as an effective, low-maintenance post-treatment step to remove residual organics.

Low percentages of removed carbon assimilated as biomass are beneficial to the long-term operation of BAC filters with low maintenance. Less biomass growing in the BAC filter leads to less clogging. We showed that a simple backwash with water alone is effective in reducing pressure loss. The loss of pressure was slow, so frequent backwashing was not necessary. Generally, the maintenance of the BAC filter with backwashing is much less than that for the MBR, where the membranes need to be exchanged and chemically cleaned.

## 5 Conclusions

This study used a BAC filter as post-treatment after an MBR and reached three main conclusions:

- Biodegradation was the main removal mechanism in the upper part of the BAC filter, whereas sorption capacity remained towards the bottom of the filter thanks to a generous filter design with low filtration rate (0.42 m/h) and long average EBCT (78 min). This design allows stable removal of organic carbon over a wide range of influent concentrations and flow rates.
- Estimations based on mass balances with long-term TOC data showed that overall, sorption and biodegradation contributed similar proportions to TOC removal.
- A BAC filter installed as post-treatment after an MBR is simple to operate, low cost, and improves the biological stability of reclaimed greywater.

**Supporting Information and additional data**: Supplementary information to this article is available online. The data presented in this publication are available at https://doi.org/10.25678/0004m0.

## Declaration of Competing Interest

The authors declare that they have no known competing financial interests or personal relationships that could have appeared to influence the work reported in this paper.

## Appendix Supplementary materials

Image, application 1

Image, application 2
