# Supplementary material for: Biological activated carbon filter for greywater post-treatment: Long-term TOC removal with adsorption and biodegradation
Source: Water Res X. 2021 Aug 13;13:100113. doi: 10.1016/j.wroa.2021.100113 (PMC8476437; doi:10.1016/j.wroa.2021.100113)
Supplement: Supplementary file 1 [file mmc1.docx]

Supporting Information for:

Biological activated carbon filter for greywater post-treatment: Long-term TOC removal with adsorption and biodegradation

*Angelika Hess^a,b^ and Eberhard Morgenroth^a,b,*^*

*^a^: Eawag: Swiss Federal Institute of Aquatic Science and Technology, 8600, Dübendorf, Switzerland*

*^b^: ETH Zürich, Institute of Environmental Engineering, 8093, Zürich, Switzerland*

**Corresponding Author. Email address: Eberhard.Morgenroth@eawag.ch*

Email contacts:

Angelika Hess: Angelika.Hess@eawag.ch

Eberhard Morgenroth: Eberhard.Morgenroth@eawag.ch

First submitted to *Water Research X* on March 27, 2021

Revised version submitted on July 18, 2021

# Comparison of TOC removal in BAC

Table S1: Comparison of removal performance for different studies looking at the removal of organic carbon in biological activated carbon filters. Some of the values are extracted from graphs and should be seen as rough estimates instead of exact values.

| Reference | EBCT  (min) | Filtration rate (m/h) | EBV treated | Mean TOC_in_  (over whole period)  (mg/L) | TOC_in_ (at the max EBV treated)^1^  (mg/L) | Influent Load ((V_treated_/M_GAC_*TOCin)  (g/kg) | TOC_out_ (at the max EBV treated)^1^  (mg/L) | TOC Removal  (%) | Water source | GAC used^7^ | Comments |
| --- | --- | --- | --- | --- | --- | --- | --- | --- | --- | --- | --- |
| This study  (first 7 cm) | 15 | 0.41 | 60’891 | 5.3 | 5.1 | 659 | 3.0 | 41 | MBR permeate (greywater) | Calgon F400 | Only 1 backwash over the whole period, periods with stagnation |
| This study  (whole BAC) | 109 | 0.41 | 8196 | 5.3 | 5.1 | 89 | 1.5 | 71 | MBR permeate (greywater) | Calgon F400 | Only 1 backwash over the whole period, periods with stagnation |
| Velten et al. (2011)  (GAC 1) | 1.65 | 8 | 100’000 | 0.96 | 0.96 | 196 | 0.9 | 6 | Ozonated water | Calgon F400 |  |
| Velten et al. (2011)  (GAC 2) | 15.76 | 5.9 | 30’000 | 1.1 | - | 72 |  | 20 | Ozonated water | Chemviron SGL 8 | No backwashing |
| Gibert et al. (2013)  (GAC A) | 13 | 7.2 | 30’000 | 3.25^2^ | - | 199 | 2.8 | 40 | Ozonated water | Calgon F400 |  |
| Gibert et al. (2013)  (GAC B) | 13 | 7.2 | 40’000 | 3.25^2^ | - | 325 | 3.0 | 20 | Ozonated water | Norit ROW 0.8 |  |
| Han et al. (2013) | 8.6^3^ | 14 | 21’000 | 2.5^4^ (COD) | 2.34 (COD) | 117 | 1.93 (COD) | 18 | Ozonated water | Not known | At the beginning: Fed with non-ozonated water |
| Ziemba et al. (2020) | 144 | - | 600 | 7.9 | 5.9 | 9.4 | 1.9 | 69 | GDM permeate (hand-washing water) | Norit 830 | No backwashing |
| Dalahmeh et al. (2014) | 960 | 5 | 3.9/174^5^ |  |  |  |  | 97 (BOD) | Artificial greywater | Not known |  |
| Zipf et al. (2016) | 144 | 0.25 | 1400 | 100 (COD) |  | 31 (COD) | 50 (COD) | 50 | Artificial greywater, pre-treated with sand filter | Not known |  |
| Fundneider et al. (2021) | 6-35^6^ | 3.7-9.7 | 40’320 | 7 | 7 | 706 |  | 20 | Advanced wastewater treatment (after membrane) | Hydraffin AR | Six different filters, looked also at EBCT, backwashing |

^1^ average of the 10 last data points.

^2^ range for influent TOC given (3-3.5 mg/L). Here, the mean was taken.

^3^ HRT instead of EBCT

^4^ range for influent COD given (1.89 – 3.15 mg/L). Here, 2.5 mg/L was taken.

^5^ not clear, how much water was treated. Therefore, no Load_in_ was calculated.

^6^ To calculate the EBV_treated_, an EBCT of 25 min was assumed

^7^ To calculate the Load_in_, the following GAC densities were assumed:

- Calgon F400: 440 – 540 kg/m^3^ 🡪 490 kg/m^3^
- Chemviron SGL 8: 460 kg/m^3^
- Norit ROW 0.8 Supra: 400 kg/m^3^
- Norit 830: 500 kg/m^3^
- Hydraffin AR: 400 kg/m^3^
- Average: 450 kg/m^3^ 🡪 chosen for publications with unknown GAC

# Linear Regression

Table S2: Coefficients, p-Value, and confidence interval for linear regression analysis (Equation 1) for the long-term TOC removal.

|  |  | **Coefficient** | **p-Value** | **95% confidence interval** |
| --- | --- | --- | --- | --- |
| **b_0_ (-)** | **7 cm** | 0.61 | 2.07E-33 | 0.53-0.70 |
|  | **22 cm** | 0.31 | 2.72E-23 | 0.25 – 0.36 |
|  | **37 cm** | 0.2 | 2.27E-16 | 0.16 – 0.25 |
|  | **Effluent** | 0.23 | 1.59E-17 | 0.18 – 0.27 |
| **b_1_ (y^-1^)** | **7 cm** | 0.168 | 3.00E-24 | 0.14- 0.20 |
|  | **22 cm** | 0.182 | 6.90E-46 | 0.16 – 0.20 |
|  | **37 cm** | 0.117 | 9.86E-34 | 0.10 – 0.13 |
|  | **Effluent** | 0.08 | 8.63E-18 | 0.06 – 0.10 |
| **b_2_ (L mg^-1^)** | **7 cm** | -0.022 | 2.00E-08 | -0.029 - -0.014 |
|  | **22 cm** | -0.019 | 1.66E-13 | -0.024 - -0.014 |
|  | **37 cm** | -0.016 | 2.94E-13 | -0.020 - -0.015 |
|  | **Effluent** | -0.019 | 9.19E-16 | -0.023 - -0.015 |
| **b_3_ (min^-1^)** | **7 cm** | -0.0019 | 4.20E-09 | -0.0025 - -0.0013 |
|  | **22 cm** | -0.00096 | 3.78E-06 | -0.0014 - -5.63E-04 |
|  | **37 cm** | -0.00035 | 0.04 | -6.83E-04 – -1.58E-05 |
|  | **Effluent** | -4.83E-06 | 0.98 | -3.62E-04 – 3.52E-04 |

# Mass balance TOC removal

**Step 1:** Estimate equilibrium loading q_e_. For this calculation assume that equilibrium loading was reached in the top 7 cm of the BAC on day 94 following the period of overloading (day 67) and subsequent desorption. Further assume during the first 94 days of operation 65% of the TOC was removed by sorption (f_sorbed_).


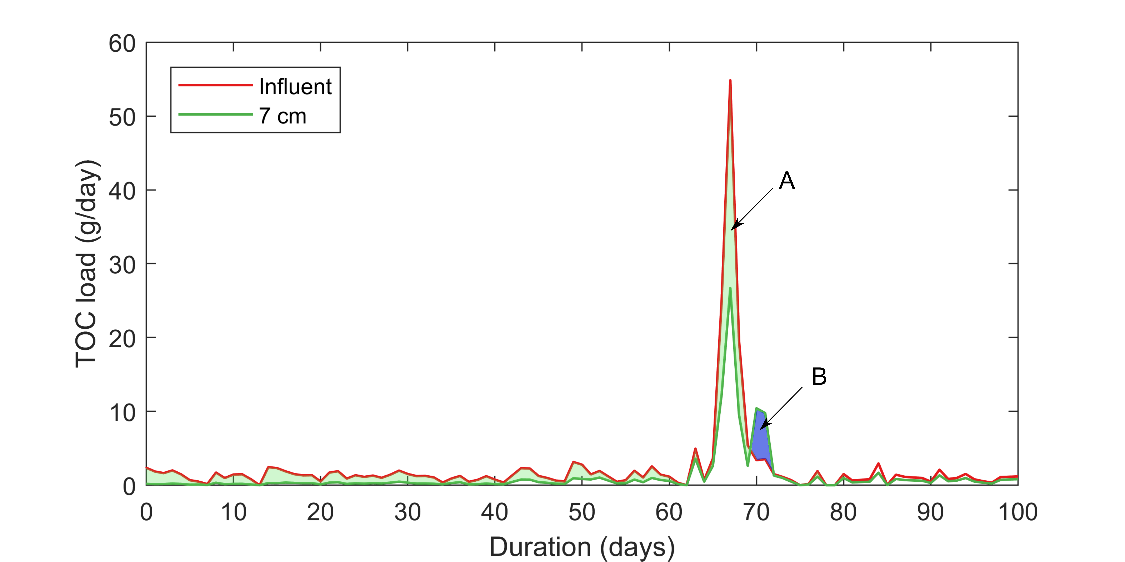


Figure S1: TOC load for the influent of the BAC filter and after 7 cm. The green shaded area (A) shows the TOV removed in the top 7 cm, and the blue shaded area (B) shows the TOC desorbed after the influent peak.

$$q_{e}=\frac{(FTOC_{Influent}-FTOC_{7cm})\cdot f_{sorbed}}{M_{GAC,7cm}}=\frac{106gTOC\cdot0.65}{2672gGAC}=25.6mgTOC/gGAC$$

**Step 2:** Calculate the amount of adsorbed TOC in the overall column from the estimated equilibrium loading (q_e_).

$$FTOC_{Sorption}=M_{GAC}\cdot q_{e}=19850g\cdot25.6mgTOC/gGAC=512gTOC$$

**Step 3:** Calculate the amount of cells that were removed with the backwash on day 836.

$$FTOC_{cells,backwash}=FTCC_{Backwash}\cdot c_{TCC}=1.1\cdot10^{12}cells\cdot2\cdot10^{-14}gTOC/cell=0.02gTOC$$

**Step 4:** Calculate the amount of cells that accumulated in the BAC filter bed based on the ATP measured after the backwashing.

$$\begin{matrix} FTOC_{cells,accumulated}=ATP\cdot c_{ATP}\cdot c_{TCC}=0.0686gATP\cdot1.1\cdot10^{14}cells/gATP\cdot2\cdot10^{-14}gTOC/cell \\ =0.15gTOC \end{matrix}$$

**Step 5:** Calculate the amount of cells that were continuously washed out from the BAC filter.

$$\begin{matrix} FTOC_{cells,washedout}=(FTCC_{Effluent}-FTCC_{Influent})\cdot c_{TCC} \\ =(2.3\cdot10^{14}cells-8.9\cdot10^{13}cells)\cdot2\cdot10^{-14}gTOC/cell \\ =2.9gTOC \end{matrix}$$

**Step 6:** Calculate the assimilated biomass as the sum of the cells that accumulated in the filter, were continuously washed out and removed with the backwashing.

$$\begin{matrix} FTOC_{assimilated}=FTOC_{cells,accumulated}+FTOC_{cells,washedout}+FTOC_{cells,backwash} \\ =0.15gTOC+2.9gTOC+0.02gTOC \\ =3.07gTOC \end{matrix}$$

**Step 7**: Estimate the amount of produced CO2 based on the biodegraded TOC and the amount of TOC that was assimilated as biomass.

$$FTOC_{CO_{2}}=FTOC_{biodegraded}-FTOC_{assimilated}=512gTOC-3.07gTOC=509gTOC$$

# TOC measurements


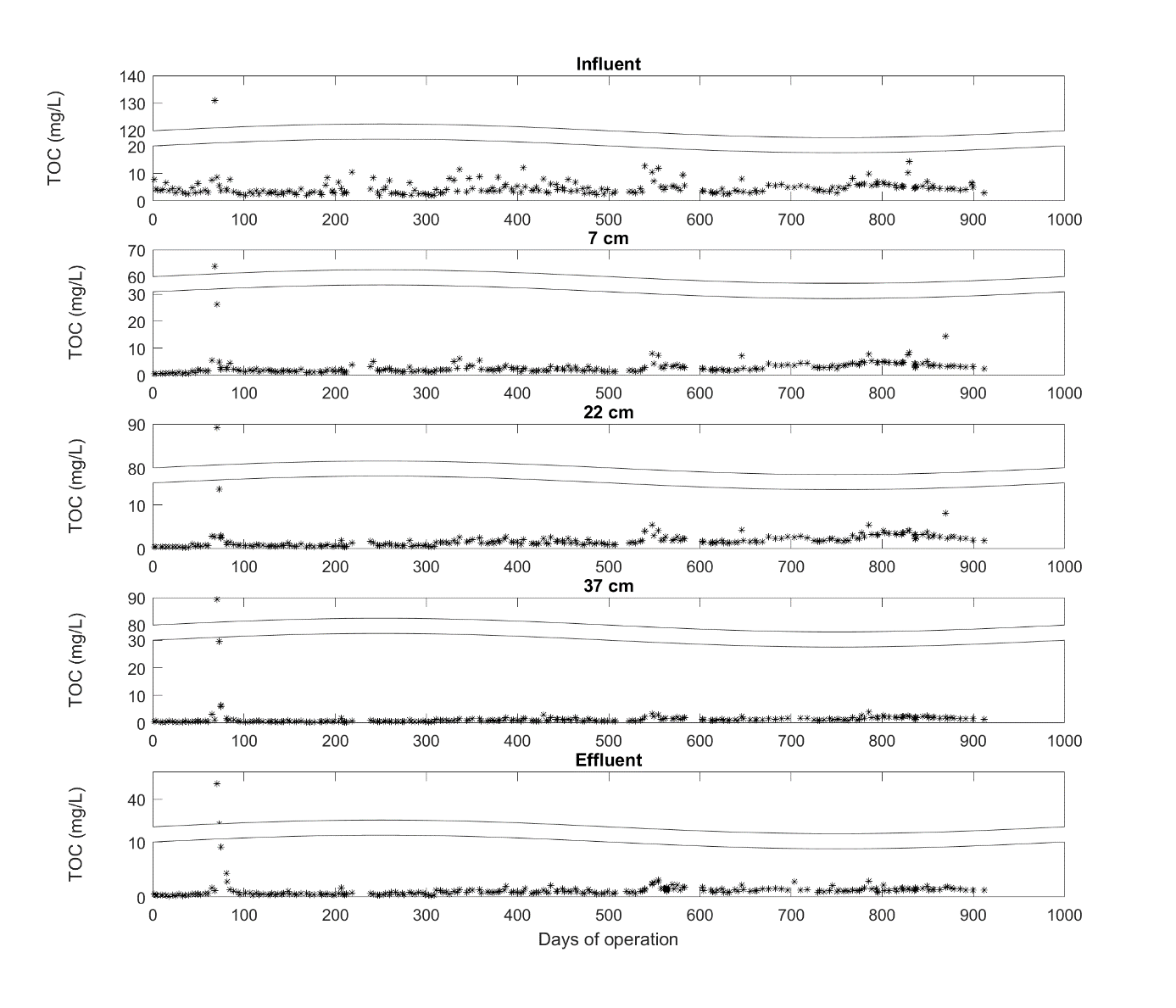


Figure S2: Absolute TOC values over time for the five measurement locations for the BAC filter.

# How is the TOC removed?

The results from the TOC batch degradation tests are shown in Figure S2.

*
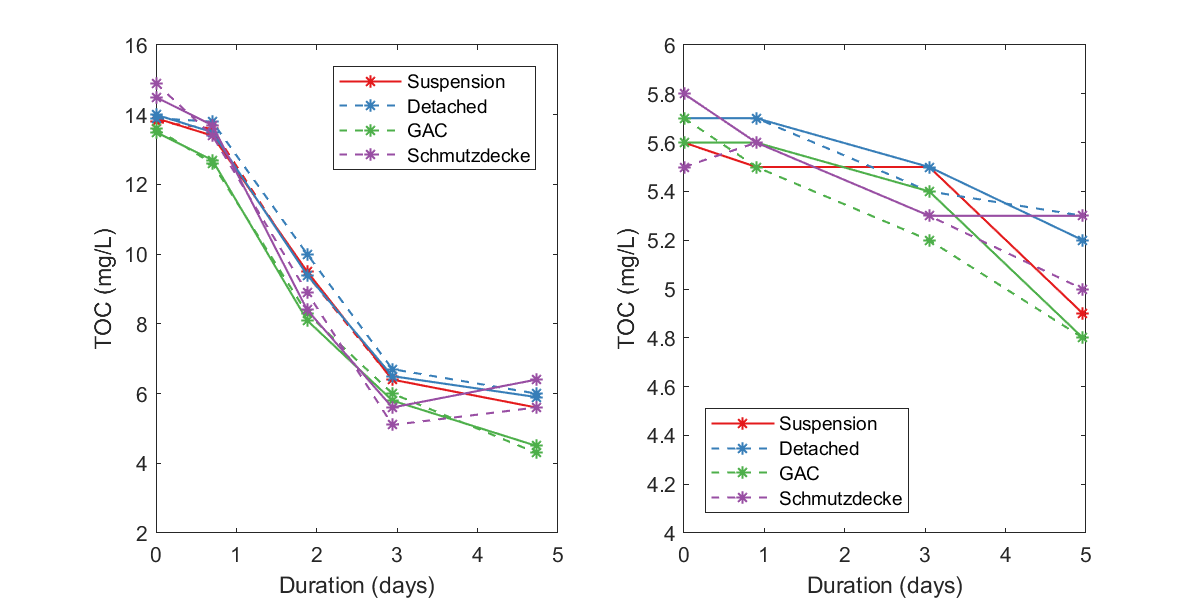
*

*Figure S3: Removed TOC in batch experiments with different biomass. For the right figure, in the full-scale BAC, 0.9 mg/L TOC were removed in the top segment.*

# Backwashing

On day 836, a backwashing was performed. Figure S3 shows the influence of the backwashing on the ATP on the GAC. Figures S4-S6 show the influence of the backwashing on the removal performance in the BAC.


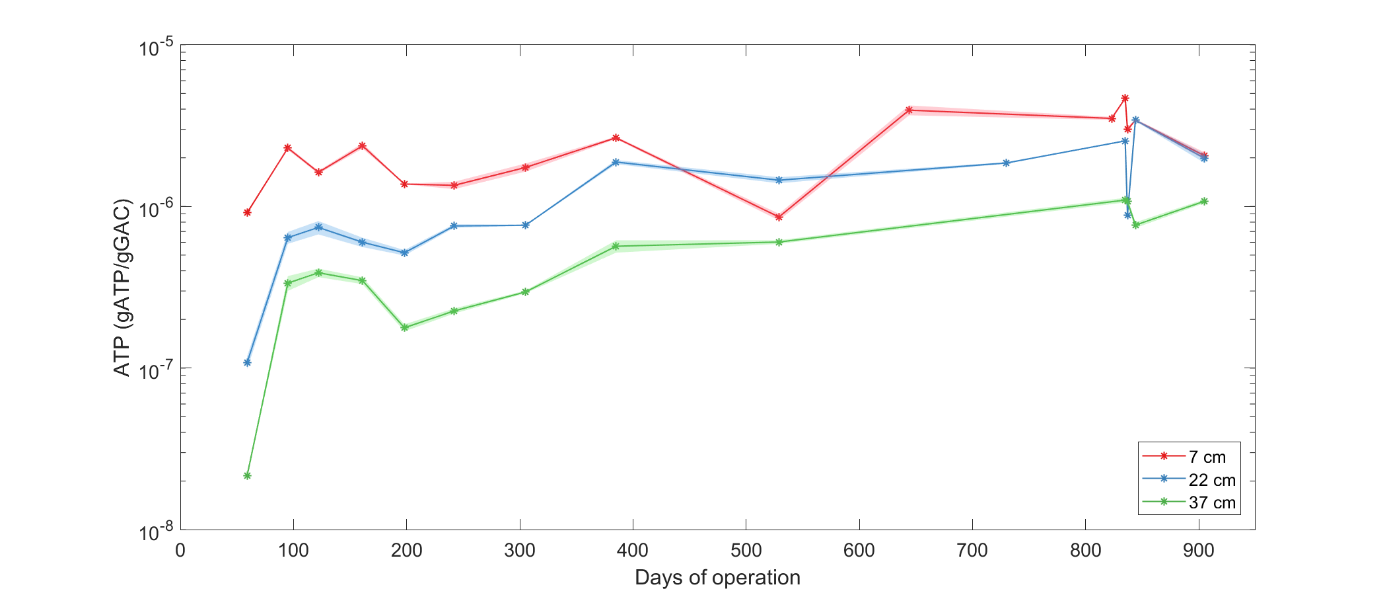


Figure S4. Measured ATP on the GAC over time for different heights of the filter bed.


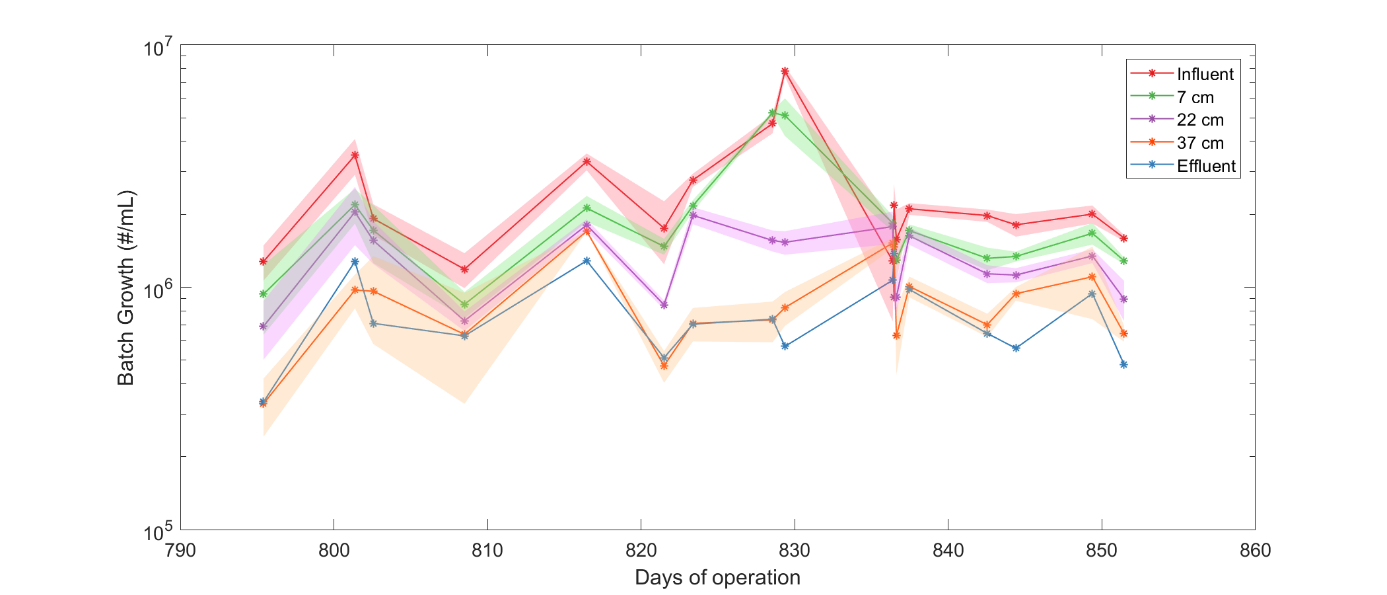


Figure S5. Batch growth measured in the influent and the effluent of the BAC before and after the backwash. The bands show the standard deviation.


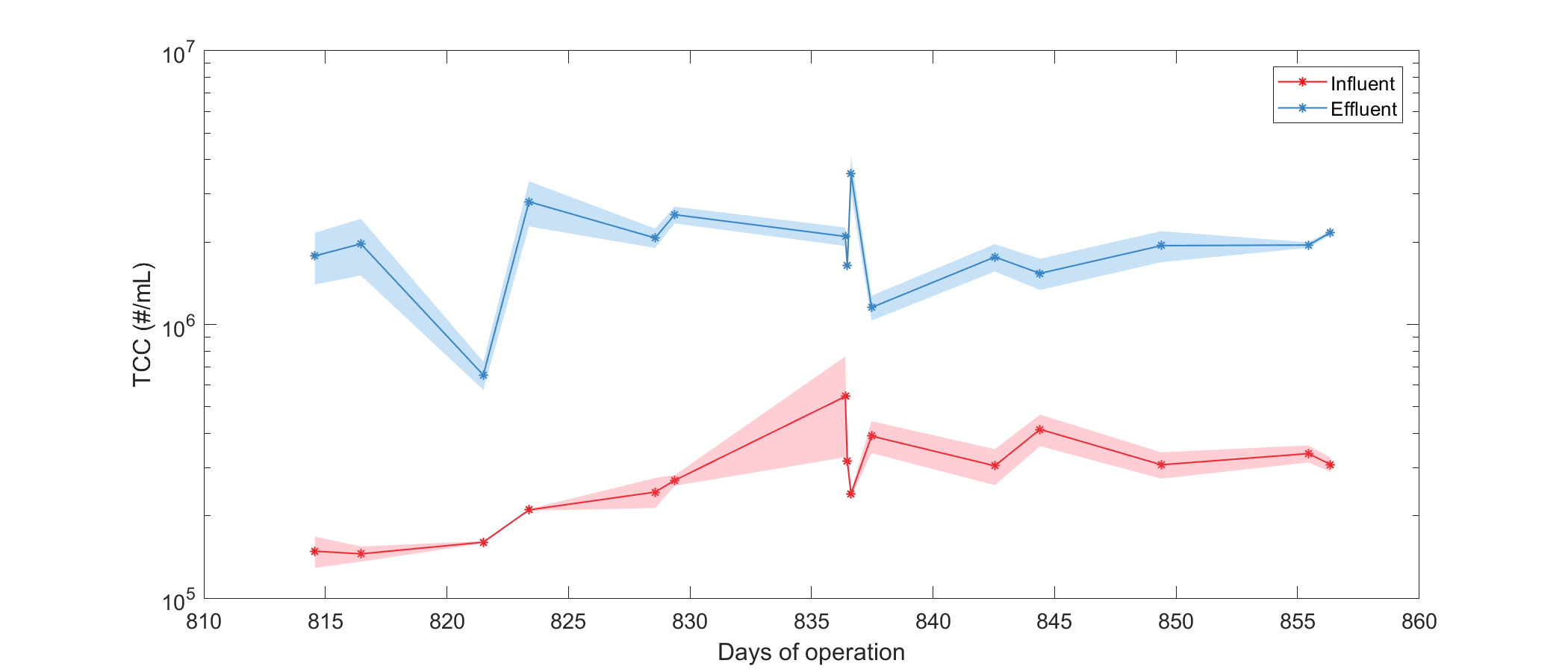


Figure S6: Total cell concentration in the influent and the effluent of the BAC before and after the backwash. The bands show the standard deviation.


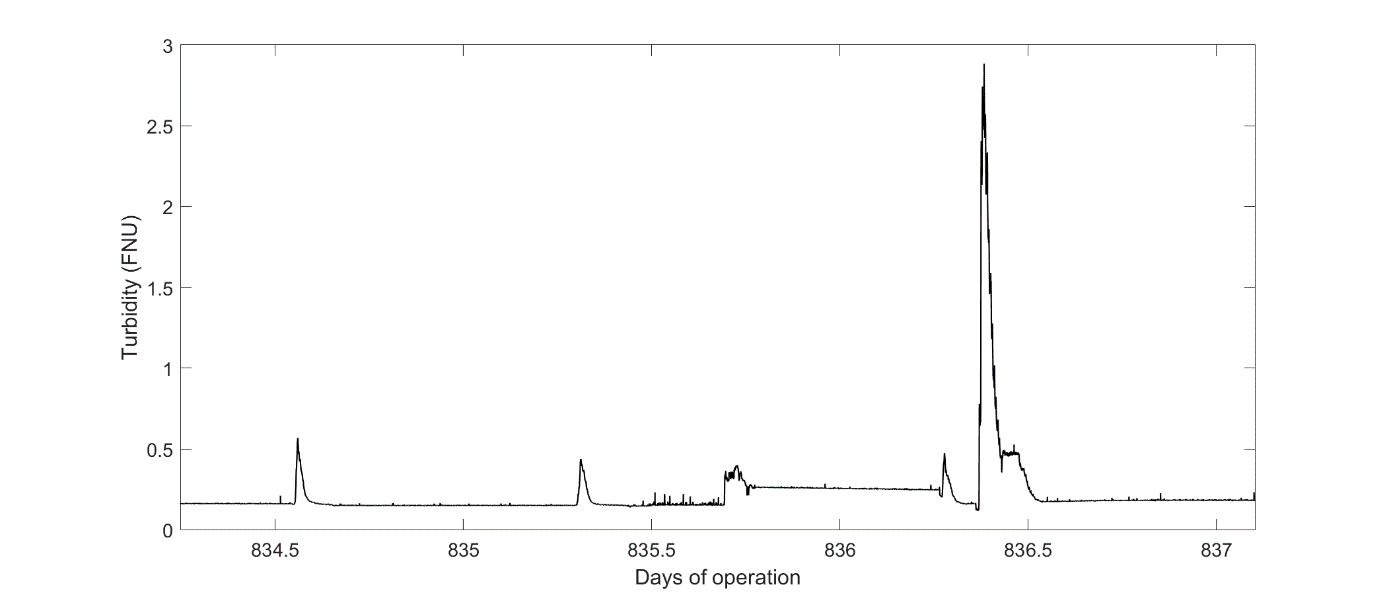


Figure S7: Turbidity measured in the effluent of the BAC before and after the backwash on day 836.4.

# Sorption capacity

Sorption capacity in the BAC was characterized with methylene blue as described in Hess et al. (2020).


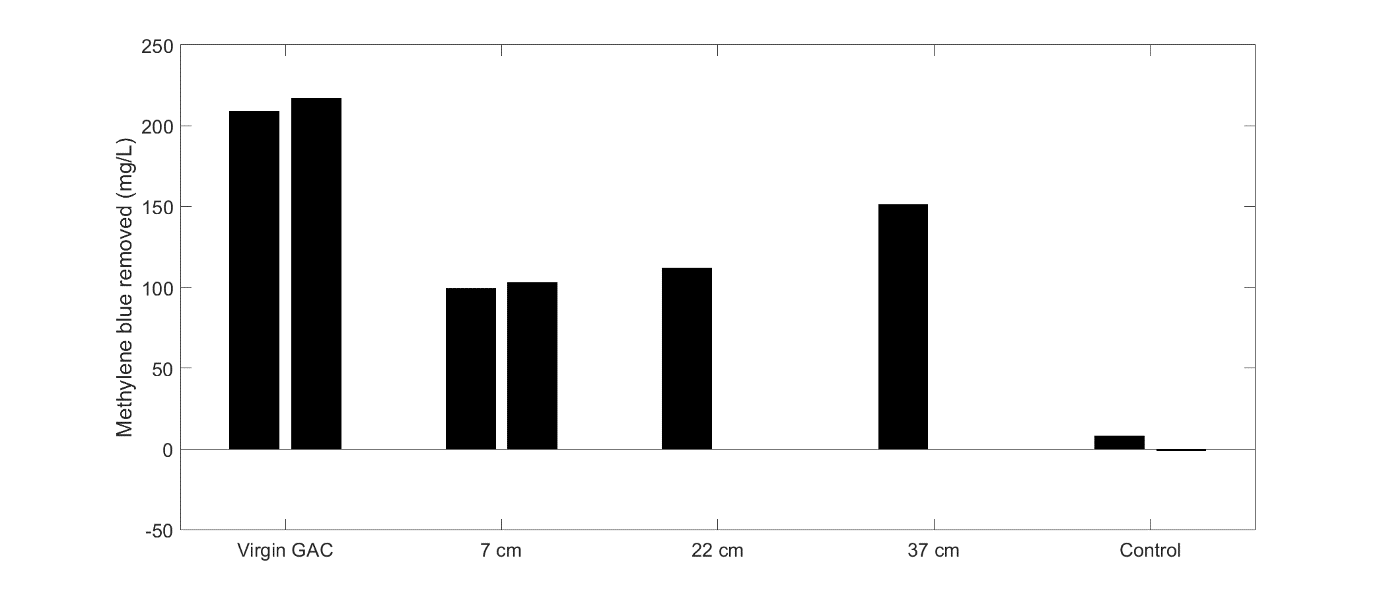


Figure S8: Methylene blue removed in batch tests to characterize the sorption capacity in the full-scale BAC filter for greywater treatment.

# References

Dalahmeh, S.S., Jönsson, H., Hylander, L.D., Hui, N., Yu, D. and Pell, M. (2014) Dynamics and functions of bacterial communities in bark, charcoal and sand filters treating greywater. Water Research 54, 21-32.

Fundneider, T., Acevedo Alonso, V., Wick, A., Albrecht, D. and Lackner, S. (2021) Implications of biological activated carbon filters for micropollutant removal in wastewater treatment. Water Research 189, 116588.

Gibert, O., Lefèvre, B., Fernández, M., Bernat, X., Paraira, M. and Pons, M. (2013) Fractionation and removal of dissolved organic carbon in a full-scale granular activated carbon filter used for drinking water production. Water Research 47(8), 2821-2829.

Han, L., Liu, W., Chen, M., Zhang, M., Liu, S., Sun, R. and Fei, X. (2013) Comparison of NOM removal and microbial properties in up-flow/down-flow BAC filter. Water Research 47(14), 4861-4868.

Hess, A., Bettex, C. and Morgenroth, E. (2020) Influence of intermittent flow on removal of organics in a biological activated carbon filter (BAC) used as post-treatment for greywater. Water Research X 9, 100078.

Velten, S., Knappe, D.R.U., Traber, J., Kaiser, H.-P., von Gunten, U., Boller, M. and Meylan, S. (2011) Characterization of natural organic matter adsorption in granular activated carbon adsorbers. Water Research 45(13), 3951-3959.

Ziemba, C., Larivé, O., Reynaert, E., Huisman, T. and Morgenroth, E. (2020) Linking transformations of organic carbon to post-treatment performance in a biological water recycling system. Science of The Total Environment 721, 137489.

Zipf, M.S., Pinheiro, I.G. and Conegero, M.G. (2016) Simplified greywater treatment systems: Slow filters of sand and slate waste followed by granular activated carbon. Journal of Environmental Management 176, 119-127.
